# Impact on hospitalization rates as a result of the implementation of a care programme for severe asthma patients in Espirito Santo, Brazil

**DOI:** 10.1186/1939-4551-8-S1-A224

**Published:** 2015-04-08

**Authors:** Lyvia Barbosa Alves, Maria José Sartório, Faradiba Serpa, Rayane Fontoura Koch, Braga Neto, Fernanda Lugão Campinhos, Marina Gaburro Silveira

**Affiliations:** 1 Escola Superior De Ciências Da Santa Casa De Misericórdia De Vitória, Brazil; 2 Secretaria Estadual De Saúde Do Espírito Santo, Brazil

## Background

To assess the rate of hospitalization before and after actions taken in the care of severe asthma patients receiving treatment through the Unified Health System.

## Methods

From 2003 to 2009 the following actions were implemented by the local health system: training of health professionals; creation of a referral outpatient clinic with a multidisciplinary team and free pharmacological services. The number of asthma related hospitalizations was collected in the period from 2002 to 2013 and rates and percentages of hospitalizations were calculated and linear regression model performed to determine the trend over this period of time. Data concerning the dispensing of medications which are included in State List of Exceptional Medicines (budesonide + formoterol 12/400 mcg) and in the State Ordinance 054-R, 12/05/2009 (salmeterol + fluticasone 25/125 and 50 / 250, 200 mcg budesonide, montelukast 4, 5 and 10 mg and omalizumab 150 mg) were also evaluated.

## Results

In the period from 2004 to 2013, 2.600 patients with severe asthma were enrolled in the program and had access to treatment. Of these, 209 (13%) required 1.600 mcg of budesonide for asthma control and 43 (1.6%) were diagnosed with difficult to control asthma and initiated anti-IgE therapy with omalizumab. Over time there was a decreasing trend in the rate and percentage of hospitalizations for asthma. There was a 72% reduction in hospitalization rates which decreased from 1.6 in 2002 to 0.45 in 2013 (R^2^ = 0.84). A reduction in the percentage of hospitalization from 2.58% in 2002 to 0.81% in 2013 (R^2^ = 0.91) was observed.

## Conclusions

The implementation of a program for severe asthma patients has allowed access to specialized care and provided medications for all steps of the treatment of asthma. The actions taken have resulted in a reduction in the rate and percentage of asthma related hospitalization.

